# Dataset of polyoxometalate-assisted *N*-heterocyclic carbene gold(I) complexes

**DOI:** 10.1016/j.dib.2019.104002

**Published:** 2019-05-24

**Authors:** Kenji Nomiya, Yuichi Murata, Yuta Iwasaki, Hidekazu Arai, Takuya Yoshida, Noriko Chikaraishi Kasuga, Toshiaki Matsubara

**Affiliations:** Department of Chemistry, Faculty of Science, Kanagawa University, Hiratsuka, Kanagawa 259-1293, Japan

**Keywords:** Polyoxometalate, *N*-Heterocyclic carbene ligand, Gold(I) complex, Homogeneous catalysis, Hydration of diphenylacetylene

## Abstract

The present paper is the Supplemental materials for our original paper entitled “highly active, homogeneous catalysis by polyoxometalate-assisted *N*-heterocyclic carbene gold(I) complexes for hydration of diphenylacetylene. The present article refers to the preparations of several monomeric, *N*-heterocyclic (NHC) carbene/carboxylate (*RS*-pyrrld)/gold(I) complexes, [Au(*RS*-pyrrld)(NHC)] (NHC = IMes (**6**), BIPr (**7**), IF^3^ (**8**), I^t^Bu (**9**)), which were used for homogenous catalysis of the hydration reaction of diphenylacetylene to afford deoxybenzoin. The article also includes the preparations of the precursor complexes, [AuCl(NHC)] (NHC = IPr, IMes, BIPr, IF^3^, I^t^Bu), and novel X-ray crystallography of the separately prepared [Au(IPr)(H_2_O)]_3_[α-PW_12_O_40_]·7Et_2_O (**2**), summary of crystal data of (**2**), and selected bond distances (Å) and angles (deg) of (**2**). Also presented are Cartesian coordinates of the optimized structures in the quantum-mechanical calculations.

**Table:** Specifications table

Subject area	*Catalysis, Inorganic Chemistry*
More specific subject area	Polyoxometalate, *N*-Heterocyclic carbene ligand, Gold(I) complex, Homogeneous catalysis, Hydration of diphenylacetylene
Type of data	Text files describing synthesis and tables
How data was acquired	*NMR and single-crystal X-ray;* The ^1^H NMR (400 MHz),^31^P{^1^H} NMR (161 MHz) and^13^C{^1^H} NMR (99 MHz) spectra of the samples were recorded in 5-mm-outer-diameter tubes on a JEOL JNM-ECA 400 FT-NMR or a JEOL JNM-ECS-400 FT-NMR spectrometer and a JEOL ECA-400 NMR or ECS-400 NMR data processing system, respectively. Single crystals of the metal complex were mounted on a loop and used for measurements of cell constants and for the collection of intensity data on a Rigaku VariMax with Saturn CCD diffractometer. The structure was solved by a direct method, followed by difference Fourier calculation; it was refined by a full-matrix least-squares method on *F*^2^ using the Yadokari program package.
Data format	Docx created by word2013
Experimental factors	Preparation, X-ray crystallography and quantum-mechanical calculations
Experimental features	▪ Preparation of Au(I) complexes ([AuCl(NHC)] complexes (NHC = IPr, IMes, BIPr, IF^3^, I^t^Bu), Preparation of [Au (*RS*-pyrrld)(NHC)] complexes (NHC = IMes (**6**), BIPr (**7**), IF^3^ (**8**), I^t^Bu (**9**))). ▪ X-ray crystallography of [Au(IPr)(H_2_O)]_3_ [α-PW_12_O_40_]·7Et_2_O (**2**) including Summary of crystal data (Table 1), Selected bond distances (Å) and angles (deg) (Table 2) and Cartesian coordinates (in Å) (Table 3). ▪ The ^1^H NMR and IR spectra of [Au (*RS*-pyrrld)(IPr)] (**1**) and ^1^H NMR of [Au(H_2_O)(IPr)]_3_ [α-PW_12_O_40_]·7Et_2_O (**2**) are shown in Fig. 1, Fig. 2, Fig. 3, respectively.
Data source location	Department of Chemistry, Faculty of Science, Kanagawa University, Hiratsuka, Kanagawa 259–1293, Japan
Data accessibility	Data are available within this article.
Related research article	▪ H. Arai, T. Yoshida, E. Nagashima, A. Hatayama, S. Horie, S. Matsunaga, K. Nomiya, Organometallics 35 (2016) 1658–1666. ▪ K. Nomiya, Y. Murata, Y. Iwasaki, H. Arai, T. Yoshida, N. C. Kasuga, T. Matsubara, Mol. Catal. 469 (2019) 144–154 [1]. ▪ F. Sirindil, S. P. Nolan, S. Dagorne, P. Pale, A. Blanc, P. de Frémont, Chem. Eur. J. 24 (2018) 12630–12637 [2]

**Table:** 

**Value of the data** • The data in this article will be informative for researchers who work on the chemistry of gold-polyoxometalate hybrids. • The data in this article will be useful for design of more active catalytic systems for alkyne hydration in the presence of polyoxometalates. • Details of synthesis and characterization of N-heterocyclic carbene (NHC)-gold(I) complexes, [AuCl(NHC)], will be informative for synthesis of other related gold(I) complexes. • The synthetic process of the catalytically active complexes, [Au(*RS*-pyrrld)(IPr)] (**1**) and [Au(*RS*-pyrrld)(NHC)] ((**6**)–(**9**)), using [AuCl(NHC)], will be applicable for other active gold(I) complexes. • The data of X-ray molecular structure of [Au(IPr)(H_2_O)]_3_[α-PW_12_O_40_] (**2**) as the actual catalyst precursor will be interesting for researchers on the chemistry of gold-polyoxometalate hybrids.

## 1 Data

**Fig. 1:**
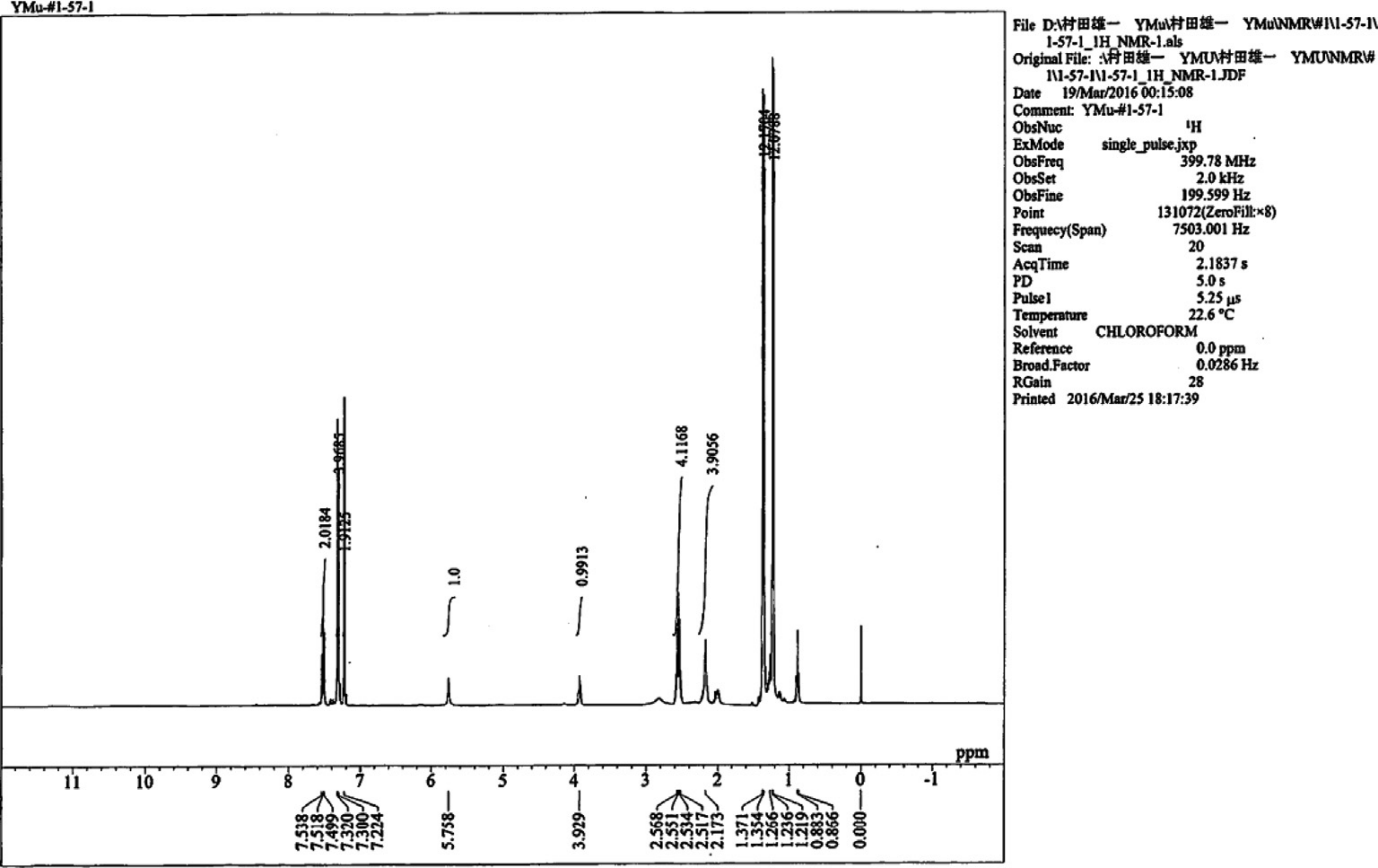
^1^H NMR spectrum of [Au(*RS*-pyrrld)(IPr)] (**1**) in CDCl_3_ at 22.6 °C.

**Fig. 2:**
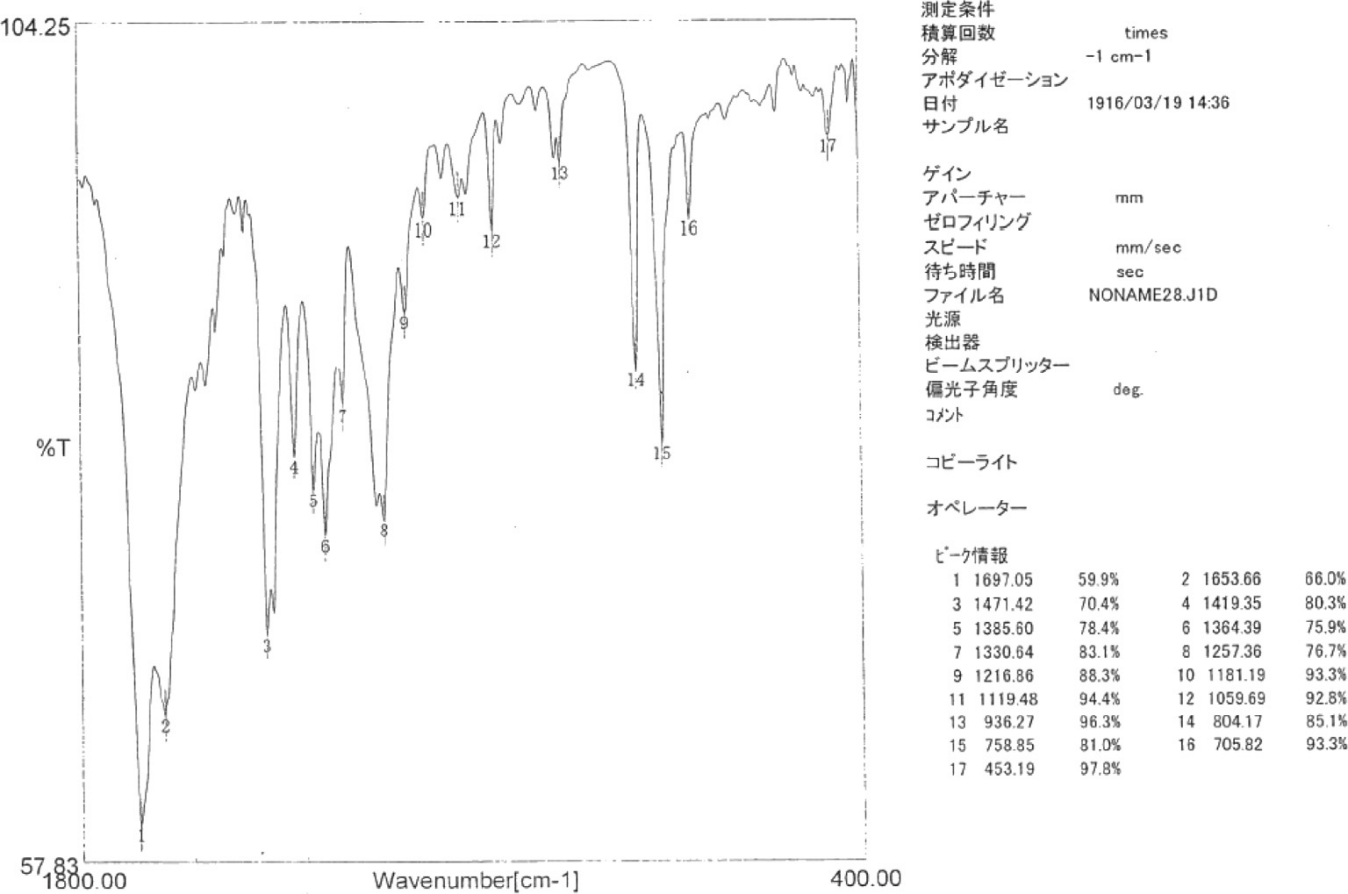
IR spectrum of [Au(*RS*-pyrrld)(IPr)] (**1**).

**Fig. 3:**
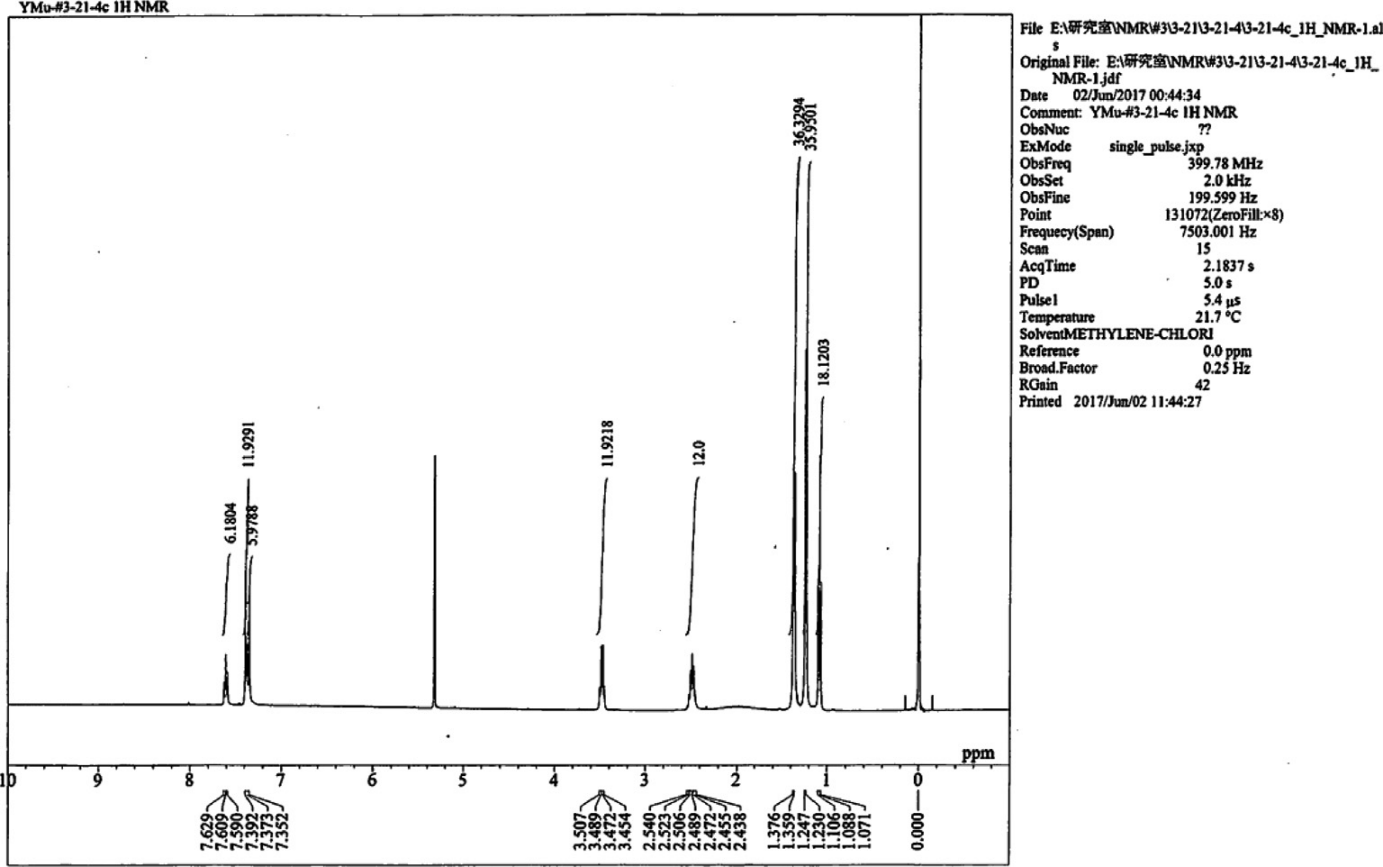
^1^H NMR spectrum of [Au(H_2_O)(IPr)]_3_[α.PW_12_O_40_] (**2**) in CD_2_Cl_2_ at 21.7 °C.

Data presented in this article displays the preparations of several precursors used for homogenous catalysis of the hydration reaction of diphenylacetylene to afford deoxybenzoin; monomeric, *N*-heterocyclic (NHC) carbene/carboxylato/gold(I) complexes, [Au(*RS*-pyrrld)(NHC)] (NHC = IMes (**6**), BIPr (**7**), IF^3^ (**8**), I^t^Bu (**9**)), as well as the precursor complexes, [AuCl(NHC)] (NHC = IPr, IMes, BIPr, IF^3^, I^t^Bu) [1]. Also presented are summary of crystal data of the separately prepared [Au(IPr)(H_2_O)]_3_[α-PW_12_O_40_]·7Et_2_O (**2**) (Table 1), selected bond distances (Å) and angles (deg) of (**2**) (Table 2), and Cartesian coordinates of the optimized structures in the quantum-mechanical calculations (Table 3). The ^1^H NMR and IR spectra of [Au (*RS*-pyrrld)(IPr)] (**1**) and ^1^H NMR spectrum of [Au(H_2_O)(IPr)]_3_ [α-PW_12_O_40_]·7Et_2_O (**2**) are shown in Fig. 1, Fig. 2, Fig. 3, respectively.

**Table 1:** Summary of crystal data of [Au(H_2_O)(IPr)]_3_[α-PW_12_O_40_]·7Et_2_O (**2**).

Empirical formula	C_109_H_178_ Au_3_N_6_O_50_PW_12_
Formula weight	1268.75
Crystal system	Orthorhombic
Space group	*P2*_*1*_*2*_*1*_*2*_*1*_ (No.19)
*a*/Å	21.4563 (2)
*b*/Å	21.6021 (2)
*c*/Å	31.3646 (3)
*α*/°	90
*β*/°	90
*γ*/°	90
*V*/Å^3^	14537.5 (2)
*D*_calcd_/g·cm^−3^	2.376
*Z*	4
*μ*/mm^−1^	12.553
*T*/K	100
No. of reflections	
Total	196846
Unique	33392
No. of observations	
(*I* > 2σ(*I*))	32699
*R*_int_	0.0595
*R1* (*I* > 2σ(*I*))	0.0246
w*R*_2_ (*I* > 2σ(*I*))	0.0626
GOF	1.068

*R1* = Σ{|Fo|-|Fc|}/Σ|Fo|, w*R*_*2*_ = [Σω(|Fo|-|Fc|)^2^/ΣωFo^2^]^1/2^, GOF = [Σω(|Fo|-|Fc|)^2^/(m-n)]^1/2^ m; No. of reflections, n; No. of parameters.

**Table 2:** Selected bond distances (Å) and angles (deg) of [Au(H_2_O)(IPr)]_3_[α-PW_12_O_40_]·7Et_2_O (**2**).

Au (1)–O (41) (H_2_O)	2.068 (5)
Au (1)–C (1)	1.948 (7)
Au (2)–O (42) (H_2_O)	2.062 (5)
Au (2)–C (28)	1.936 (5)
Au (3)–O (43) (H_2_O)	2.071 (5)
Au (3)–C (55)	1.955 (6)
C (1)-Au (1)-O (41)	177.5 (3)
C (28)-Au (2)-O (42)	179.2 (3)
C (55)-Au (3)-O (43)	177.5 (3)
O (41) … O (44)^i^	2.526 (14)
O (41) … O (45)	2.602 (11)
O (42) … O (47)	2.657 (10)
O (42) … O (49)	2.581 (10)
O (43) … O (46) ^ii^	2.606 (10)
O (43) … O (48)^ii^	2.641 (11)

Symmetry operations; i = -x, 0.5 + y,0.5-z. ii = 1-x, −0.5 + y, 0.5-z.

**Table 3:** Cartesian coordinates (in Å).

[(IPr)Au]^+^
Au −0.000097–0.000050 -1.609283
C −0.000038 0.000136 0.381573
N −1.078422–0.026872 1.181125
N 1.078389 0.027269 1.181062
C −0.679079–0.022403 2.504786
C 0.679117 0.022963 2.504745
H −1.398147–0.048544 3.309907
H 1.398229 0.049211 3.309822
C −2.441449–0.103760 0.709816
C 2.441407 0.103860 0.709673
C −3.187295 1.084250 0.650852
C −4.489906 0.982350 0.159775
C −5.008357–0.244514 -0.248124
C −4.238808–1.399557 -0.167035
C −2.928722–1.358232 0.319063
C −2.595226 2.427872 1.047910
H −5.109623 1.871079 0.095716
H −6.024449–0.299816 -0.626590
H −4.663446–2.349385 -0.480186
C −2.108410–2.634645 0.416430
C 3.186994–1.084311 0.650703
C 4.489642–0.982690 0.159657
C 5.008379 0.244064–0.248191
C 4.239093 1.399284–0.167063
C 2.928985 1.358240 0.318987
C 2.594708–2.427814 1.047838
H 5.109158–1.871561 0.095603
H 6.024496 0.299154–0.626621
H 4.663956 2.349026–0.480168
C 2.108942 2.634827 0.416314
C −3.616336 3.350312 1.721972
H −1.791182 2.250272 1.772222
C −1.976001 3.120627–0.175859
H −1.537478 4.082515 0.111330
H −1.186685 2.510648–0.632392
H −2.741420 3.307319–0.937472
H −3.108415 4.231141 2.125851
H −4.367999 3.709199 1.011537
H −4.135088 2.846603 2.543189
C 3.615436–3.349967 1.722877
H 1.790199–2.249997 1.771583
C 1.976287–3.121024 -0.176074
H 3.107307–4.230677 2.126757
H 4.367563–3.709074 1.013044
H 4.133655–2.845934 2.544230
H 1.537533–4.082785 0.111194
H 1.187301–2.511189 -0.633368
H 2.742194–3.308043 -0.937116
H 1.118591 2.391084 0.821335
C 2.763627 3.633200 1.380600
C 1.899484 3.265971–0.965724
H −1.118151–2.390691 0.821552
C −2.762990–3.633155 1.380652
C −1.898642–3.265736 -0.965579
H 2.138686 4.526018 1.482985
H 2.903322 3.193781 2.373277
H 3.744427 3.952358 1.013053
H 1.414537 2.563718–1.656166
H 1.268567 4.157118–0.887434
H 2.852011 3.567478–1.414150
H −2.137878–4.525848 1.483099
H −2.902884–3.193773 2.373318
H −3.743687–3.952498 1.012988
H −1.413774–2.563371 -1.655964
H −1.267525–4.156734 -0.887208
H −2.851033–3.567483 -1.414128
[(IPr)Au(C_2_Ph_2_)]^+^
Au 0.000394 0.588216 0.000911
C −0.000726–1.418942 -0.001712
N −1.034455–2.231477 0.286980
N 1.032137–2.231779 -0.292623
C −0.657220–3.557563 0.173318
C 0.653501–3.557764 -0.182669
H −1.352486–4.362269 0.359063
H 1.347876–4.362713 -0.370699
C −2.351525–1.757271 0.631542
C 2.349801–1.757922 -0.635384
C −2.688036–1.673634 1.992411
C −3.962319–1.191428 2.298847
C −4.848842–0.820320 1.290516
C −4.477396–0.914431 -0.045710
C −3.212023–1.384198 -0.409990
C −1.701504–2.041640 3.090288
H −4.271128–1.111605 3.336473
H −5.840081–0.460941 1.552873
H −5.179062–0.617055 -0.821001
C −2.827579–1.478134 -1.878091
C 2.686669–1.669945 -1.995862
C 3.961396–1.187659 -2.300387
C 4.848086–0.820898 -1.290614
C 4.476415–0.919735 0.045214
C 3.210428–1.389175 0.407599
C 1.700071–2.033438 -3.095185
H 4.270377–1.104336 -3.337690
H 5.839679–0.461433 -1.551504
H 5.178184–0.625608 0.821642
C 2.825197–1.486450 1.875242
C −2.372820–2.727964 4.284487
H −0.973862–2.748899 2.675233
C −0.928628–0.796964 3.553105
H −0.215474–1.063299 4.340994
H −0.370256–0.335500 2.730188
H −1.618798–0.047133 3.956924
H −1.609031–3.102590 4.972429
H −3.002257–2.032807 4.849641
H −2.994122–3.571726 3.968872
C 2.371089–2.716929 -4.291190
H 0.971462–2.741085 -2.682490
C 0.928843–0.786489 -3.554629
H 1.607162–3.088296 -4.980746
H 3.001835–2.020824 -4.853718
H 2.991031–3.562550 -3.977889
H 0.216129–1.049529 -4.344012
H 0.370174–0.327096 -2.730749
H 1.620124–0.036068 -3.955457
H 1.809242–1.894751 1.947010
C 3.759436–2.445475 2.624706
C 2.815110–0.101394 2.535027
H −1.811850–1.886718 -1.951298
C −3.762629–2.435282 -2.628974
C −2.817142–0.091800 -2.535081
H 3.431120–2.557944 3.662873
H 3.772055–3.435770 2.158090
H 4.787119–2.067271 2.639675
H 2.096488 0.568785 2.048531
H 2.542281–0.187313 3.592121
H 3.800789 0.374373 2.476586
H −3.435667–2.544857 -3.667878
H −3.774419–3.426845 -2.165022
H −4.790405–2.057245 -2.641480
H −2.098242 0.577166–2.047347
H −2.544380–0.175796 -3.592344
H −3.802620 0.384264–2.475766
C 0.608991 2.829050 0.107363
C −0.606506 2.829697–0.099756
C 1.998472 3.030917 0.441925
C −1.995643 3.033171–0.434768
C 3.011243 2.339389–0.239229
C 4.341359 2.565489 0.098132
C 4.665875 3.463803 1.114469
C 3.657522 4.141093 1.801368
C 2.324008 3.929631 1.468544
H 2.755132 1.627085–1.022333
H 5.125698 2.038342–0.435704
H 5.706194 3.638278 1.371715
H 3.909847 4.837412 2.594773
H 1.531113 4.452813 1.993776
C −2.319878 3.935453–1.458679
C −3.652941 4.147914–1.792655
C −4.662168 3.467812–1.109818
C −4.338907 2.565864–0.096320
C −3.009274 2.339077 0.242449
H −1.526316 4.460446–1.981090
H −3.904190 4.847081–2.583902
H −5.702138 3.642794–1.368129
H −5.123774 2.035845 0.433875
H −2.754289 1.623855 1.023281
[(IPr)Au(H_2_O)]^+^
Au −0.000561 0.023782 1.485084
C −0.000071–0.009431 -0.492262
N −1.075279 0.007267–1.302871
N 1.074310–0.045101 -1.303227
C −0.679504–0.011174 -2.627682
C 0.677599–0.055525 -2.627871
H −1.398818 0.005115–3.432688
H 1.396256–0.089576 -3.432883
C −2.438517 0.087026–0.837800
C 2.437651–0.111578 -0.837043
C −3.186131–1.099821 -0.773965
C −4.491589–0.996174 -0.290848
C −5.013293 0.233114 0.105781
C −4.243510 1.387592 0.020338
C −2.930702 1.342981–0.458533
C −2.590681–2.445271 -1.159896
H −5.111685–1.884687 -0.225803
H −6.032130 0.290731 0.476745
H −4.670253 2.339987 0.323000
C −2.112040 2.619722–0.564557
C 3.179033 1.079542–0.786128
C 4.483845 0.988350–0.298134
C 5.011103–0.233197 0.114113
C 4.247570–1.392969 0.039984
C 2.935774–1.360394 -0.441972
C 2.580174 2.417327–1.192281
H 5.098595 1.881173–0.241653
H 6.029468–0.281084 0.487770
H 4.679090–2.339703 0.353657
C 2.122371–2.641576 -0.533495
C −3.609325–3.378644 -1.822091
H −1.789205–2.270726 -1.887492
C −1.964080–3.123329 0.068307
H −1.518041–4.083983 -0.211984
H −1.180925–2.501596 0.518750
H −2.728923–3.311101 0.830700
H −3.098925–4.262246 -2.216932
H −4.359268–3.732122 -1.106926
H −4.130791–2.885735 -2.648200
C 3.592334 3.333029–1.888579
H 1.768712 2.228705–1.905181
C 1.971501 3.123499 0.028807
H 3.078575 4.207955–2.298135
H 4.350897 3.702270–1.190691
H 4.103708 2.820245–2.708905
H 1.525166 4.079252–0.267064
H 1.191660 2.515385 0.502838
H 2.745248 3.325330 0.778386
H 1.128934–2.401844 -0.932192
C 2.777463–3.636782 -1.500470
C 1.922218–3.272486 0.850075
H −1.116128 2.369827–0.950920
C −2.756044 3.599037–1.555118
C −1.922073 3.275432 0.808940
H 2.157266–4.533405 -1.599541
H 2.909252–3.196846 -2.494028
H 3.762386–3.949933 -1.138329
H 1.432574–2.570983 1.537175
H 1.297829–4.168659 0.773861
H 2.879043–3.567727 1.294266
H −2.133011 4.492628–1.663541
H −2.880003 3.142899–2.542422
H −3.743405 3.920286–1.206928
H −1.446628 2.586865 1.518263
H −1.290400 4.165400 0.721105
H −2.881175 3.587537 1.236111
O −0.012789 0.110453 3.685545
H 0.825203–0.050442 4.147939
H −0.703174–0.386546 4.152821
[(PPh_3_)Au]^+^
Au −0.004449–0.011261 -2.044328
P −0.000622 0.001979 0.238992
C 1.153850–1.247090 0.876880
C 0.510092 1.629023 0.863995
C −1.660838–0.366992 0.877151
C 2.064052–0.850550 1.866761
C 2.956235–1.769293 2.409294
C 2.938043–3.088157 1.963519
C 2.026210–3.482676 0.987347
C 1.118606–2.583279 0.421732
H 2.075410 0.176241 2.220857
H 3.654953–1.453933 3.177218
H 3.629454–3.814465 2.379299
H 2.012069–4.516810 0.653954
H 0.429798–2.940035 -0.338310
C −0.281643 2.227023 1.854613
C 0.074406 3.461054 2.388439
C 1.226071 4.097679 1.933967
C 2.016226 3.497832 0.956137
C 1.684028 2.260537 0.398433
H −1.175452 1.726795 2.216543
H −0.542530 3.915536 3.156660
H 1.516350 5.060027 2.344120
H 2.918721 3.998195 0.615537
H 2.331696 1.836802–0.363388
C −1.774720–1.345802 1.874267
C −3.017223–1.653034 2.418385
C −4.148603–0.978488 1.967354
C −4.032088 0.000317 0.983249
C −2.799039 0.329827 0.415426
H −0.891675–1.866506 2.233269
H −3.095918–2.410238 3.191516
H −5.124141–1.208043 2.384746
H −4.919707 0.527978 0.644887
H −2.761675 1.098146–0.351180
[(PPh_3_)Au(C_2_Ph_2_)]^+^
Au 0.791400 0.070986 0.048181
P −1.479093–0.266220 -0.014319
C −1.941770–1.946346 -0.560908
C −2.253202–0.018372 1.620302
C −2.282750 0.898845–1.170516
C −2.941275–2.627848 0.145041
C −3.344124–3.900650 -0.249163
C −2.748590–4.495085 -1.357590
C −1.762083–3.813044 -2.065958
C −1.336895–2.535701 -1.690789
H −3.413815–2.162272 1.005235
H −4.121683–4.417320 0.304068
H −3.055482–5.485903 -1.678020
H −1.310312–4.278675 -2.937852
H −0.568578–2.044136 -2.280237
C −3.368034 0.822552 1.719909
C −3.992527 1.027429 2.947559
C −3.503655 0.384467 4.080437
C −2.400701–0.460854 3.979151
C −1.751820–0.680416 2.761258
H −3.757173 1.317726 0.834575
H −4.857049 1.680297 3.011210
H −3.982398 0.532015 5.043604
H −2.032568–0.969140 4.866560
H −0.898081–1.350993 2.732069
C −3.192076 0.390602–2.107469
C −3.828518 1.237700–3.010048
C −3.561969 2.602873–2.972397
C −2.668482 3.110289–2.031826
C −2.009908 2.282765–1.118342
H −3.410533–0.673052 -2.131292
H −4.531096 0.830821–3.730010
H −4.053490 3.276480–3.667739
H −2.475137 4.179376–1.999272
H −1.319247 2.733823–0.411992
C 2.936435 1.139206 0.090020
C 3.202043–0.060758 0.076396
C 2.733770 2.564921 0.068145
C 3.655100–1.427293 0.053851
C 2.416350 3.252057 1.249209
C 2.185130 4.622415 1.204766
C 2.266074 5.307927–0.008431
C 2.585221 4.624620–1.182541
C 2.817790 3.253757–1.150854
H 2.353523 2.708723 2.187605
H 1.943919 5.157352 2.117751
H 2.086041 6.378094–0.037501
H 2.655179 5.160200–2.123961
H 3.064301 2.711200–2.058392
C 3.903714–2.055471 -1.175822
C 4.363508–3.367211 -1.196449
C 4.570848–4.055433 -0.000276
C 4.318544–3.432047 1.222443
C 3.859297–2.120265 1.256046
H 3.746137–1.507974 -2.100666
H 4.565150–3.851824 -2.146433
H 4.932950–5.078499 -0.021026
H 4.486062–3.966966 2.151766
H 3.669448–1.622858 2.202588
[(PPh_3_)Au(H_2_O)]^+^
Au −1.897057–0.030759 0.053660
P 0.371416 0.005688–0.008630
C 1.029737–1.187236 -1.221609
C 1.009623 1.650952–0.472238
C 1.103585–0.417073 1.607841
C 2.002799–0.746345 -2.127549
C 2.551835–1.623393 -3.058183
C 2.133099–2.950246 -3.079198
C 1.175951–3.392073 -2.168524
C 0.603582–2.532521 -1.226979
H 2.339522 0.286232–2.107239
H 3.305104–1.270154 -3.754984
H 2.555417–3.646115 -3.797729
H 0.863772–4.432916 -2.182588
H −0.136028–2.927208 -0.536620
C 2.030187 2.212002 0.305844
C 2.562310 3.457142–0.016498
C 2.077211 4.143664–1.125311
C 1.070643 3.580253–1.906331
C 0.515802 2.333791–1.604617
H 2.417394 1.675227 1.167177
H 3.352766 3.881078 0.594149
H 2.484413 5.114895–1.388918
H 0.705116 4.117043–2.777674
H −0.263695 1.937830–2.248818
C 2.130103–1.369355 1.646276
C 2.731914–1.711028 2.854058
C 2.310799–1.094284 4.028199
C 1.299303–0.137309 3.987589
C 0.675160 0.222237 2.790487
H 2.467155–1.845820 0.730266
H 3.526649–2.449658 2.871860
H 2.773032–1.349999 4.976646
H 0.985410 0.349688 4.906980
H −0.105161 0.977276 2.810748
O −4.140905–0.086104 0.125021
H −4.632623 0.674704 0.472218
H −4.638234–0.430586 -0.633390

## 2 Experimental design, materials, and methods

CHN elemental analyses were carried out using a PerkinElmer 2400 CHNS Elemental Analyzer II (Kanagawa University). IR spectra were recorded on a Jasco 4100 FT-IR spectrometer in KBr disks at room temperature. TG/DTA was performed using a Rigaku Thermo Plus 2 series TG/DTA TG 8120 instrument.

The ^1^H NMR (400 MHz), ^31^P{^1^H} NMR (161 MHz) and ^13^C{^1^H} NMR (99 MHz) spectra of the samples were recorded in 5-mm-outer-diameter tubes on a JEOL JNM-ECA 400 FT-NMR or a JEOL JNM-ECS-400 FT-NMR spectrometer and a JEOL ECA-400 NMR or ECS-400 NMR data processing system, respectively. The ^1^H and ^13^C{^1^H} NMR spectra were referenced to an internal TMS. The ^31^P{^1^H} NMR spectra were referenced to an external standard, 25% H_3_PO_4_ in H_2_O in a sealed capillary. The ^31^P{^1^H} NMR data with the usual 85% H_3_PO_4_ reference were shifted +0.544 ppm from these data.

The high-performance liquid chromatography (HPLC) apparatus and conditions are as follows: Shimadzu LC-20AD with Shimadzu SPD-20A detector (wavelength 260 nm), column VP-ODS (150 mm × 4.6 mm), flow rate 0.7 mL per min, and solvent MeOH: water (30 : 17).

### 2.1 Preparation of [AuCl(NHC)] complexes (NHC = IPr, IMes, BIPr, IF^3^, I^t^Bu)

The [AuCl(NHC)] complexes (NHC = IPr, IMes, BIPr, IF^3^ and I^t^Bu) were prepared by reaction of H [AuCl_4_]·4H_2_O with the NHC ligands (HIPr^+^Cl^−^, HIMes^+^Cl^−^) and Na_2_CO_3_, or by reaction of [AuCl(THT)] (THT = tetrahydrothiophene) with the NHC precursors (HBIPr^+^Cl^−^, HIF^3 +^Cl^−^, HI^t^Bu^+^Cl^−^) and K_2_CO_3_, according to the cited references.

#### 2.1.1 [AuCl(IPr)] [3]

To a solution of H [AuCl_4_]·4H_2_O (1.00 g, 2.43 mmol) in 9 mL of 3-chloropyridine, HIPr^+^Cl^−^ [3] (1.03 g, 2.43 mmol) and then Na_2_CO_3_ (1.03 g, 12.2 mmol) were sequentially added. The mixture was stirred for 24 h in an oil bath at ca 80 °C, then cooled to room temperature, and 18 mL of CH_2_Cl_2_ was added. The resulting brown suspension was filtered through a folded filter paper (Whatman #5). Dichloromethane was removed from the filtrate with a rotary evaporator at ca 30 °C, and the residual solution was added to 300 mL of hexane, affording a yellow-white suspension. Filtration on a membrane filter (JV 0.1 μm) gave a yellow-white powder, which was washed with MeOH (10 mL x 2) and hexane (30 mL x 2), dried thoroughly by suction, and dried in vacuo for 2 h. Yield 0.366 g (24.3%).

Anal. Calcd for C_27_H_36_N_2_ClAu or [AuCl(IPr)]: C, 52.22; H, 5.84; N, 4.51. Found: C, 52.28; H, 6.24; N, 4.43%. TG/DTA under atmospheric conditions: a weight loss of 79.46% due to decomposition at below 500.0 °C was observed with an endothermic peak at 353.5 °C and an exothermic peak at 432.9 °C. IR (KBr, cm^−1^) [AuCl(IPr)]: 1683 (w), 1581 (w), 1550 (w), 1470 (vs), 1456 (vs), 1415 (s), 1384 (m), 1364 (m), 1327 (m), 1254 (w), 1212 (w), 1177 (w), 1116 (w), 1058 (w), 976 (vw), 937 (w), 808 (s), 764 (s), 742 (s), 705 (w), 450 (vw). ^1^H NMR (22.0 °C, CDCl_3_) [AuCl(IPr)]: *δ*_H_ 1.22 (12H, d, *J* 7.2 Hz, *H*6), 1.34 (12H, d, *J* 7.2 Hz, *H*6), 2.55 (4H, sept, *J* 6.8 Hz, *H*5), 7.12 (2H, s, *H*2), 7.28 (4H, d, *J* 7.6 Hz, *H*7), 7.50 (2H, t, *J* 7.6 Hz, *H*8). ^13^C{^1^H} NMR (22.0 °C, CDCl_3_) [AuCl(IPr)]: *δ*_C_ 24.02 (s, *C*6), 24.47 (s, *C*6), 28.78 (s, *C*5), 123.11 (s, *C*2), 124.25 (s, *C*8), 130.71 (s, *C*7), 133.97 (s, *C*4), 145.58 (s, *C*3), 175.26 (s, *C*1).

#### 2.1.2 [AuCl(IMes)] [4]

The complex [AuCl(IMes)] was prepared by reaction of a solution of H [AuCl_4_]·4H_2_O (1.00 g, 2.43 mmol) in 9 mL of 3-chloropyridine with HIMes^+^Cl^−^ [3], [4] (0.83 g, 2.43 mmol) and Na_2_CO_3_ (6.45 g, 60.9 mmol). Workup as described above for [AuCl(IPr)] [3] afforded a pale yellow powder. Yield 0.371 g (28.5%).

Anal. Calcd for C_21_H_24_N_2_ClAu or [AuCl(IMes)]: C, 46.98; H, 4.51; N, 5.22. Found: C, 46.69; H, 6.18; N, 5.23%. TG/DTA under atmospheric conditions: a weight loss of 68.10% due to decomposition at below 500.0 °C was observed with an endothermic peak at 319.9 °C and exothermic peaks at 365.7 and 438.3 °C. IR (KBr, cm^−1^): 1745 (vw), 1705 (vw), 1609 (w), 1556 (w), 1488 (vs), 1444 (m), 1414 (m), 1378 (m), 1346 (w), 1293 (w), 1234 (s), 1167 (vw), 1122 (vw), 1091 (vw), 1079 (vw), 1036 (w), 1014 (vw), 979 (vw), 963 (vw), 931 (w), 865 (s), 749 (m), 731 (w), 705 (m), 646 (w), 596 (w), 575 (m), 430 (w). ^1^H NMR (21.3 °C, CDCl_3_): *δ*_H_ 2.10 (12H, s, *H*5), 2.34 (6H, s, *H*8), 6.99 (4H, s, *H*6), 7.09 (2H, s, *H*2). ^13^C{^1^H} NMR (22.3 °C, CDCl_3_): *δ*_C_ 17.76 (*C*5), 21.15 (*C*8), 122.19 (*C*2), 129.49 (*C*6), 134.64 (*C*4 or *C*7), 134.69 (*C*4 or *C*7), 139.77 (*C*3), 173.27 (*C*1).

#### 2.1.3 [AuCl(BIPr)] [5]

The complex [AuCl(BIPr)] was prepared by reaction of HBIPr^+^Cl^−^ [6] (0.291 g, 0.613 mmol) in 60 mL of acetone with [AuCl(THT)] [7], [8] (0.271 g, 0.920 mmol) and K_2_CO_3_ (0.424 g, 3.07 mmol) in an oil bath at ca 60 °C for 2 h with stirring. The mixture was filtered through a membrane filter (JV 0.1 μm), and the filtrate was evaporated to dryness. The resulting pale purple solid was dissolved in 20 mL of CH_2_Cl_2_, and the solution was filtered through a folded filter paper (Whatman #5). The pale purple clear filtrate was added to 600 mL of hexane. Filtration on a membrane filter (JV 0.1 μm) gave a pale purple solid, which was washed with hexane (20 mL x 2), dried thoroughly by suction, and dried in vacuo for 2 h to afford a pale purple powder. Yield 0.1290 g (70.5%).

Anal. Calcd for C_31_H_38_N_2_ClAu or [AuCl(BIPr)]: C, 55.48; H, 5.71; N, 4.17. Found: C, 55.59; H, 5.68; N, 4.06%. IR (KBr, cm^−1^): 1468 (s), 1455 (s), 1392 (vs), 1360 (vs), 1254 (w), 1225 (w), 1184 (w), 1160 (w), 1061 (w), 1009 (w), 938 (w), 903 (vw), 798 (s), 754 (vs), 638 (vw), 594 (w), 430 (w), 419 (w). ^1^H NMR (21.8 °C, CDCl_3_): *δ*_H_ 1.09 (d, *J* 6.9 Hz, *H*10), 1.33 (d, *J* 7.0 Hz, *H*10), 2.40 (sept, *J* 6.9 Hz, *H*9), 7.09 (dd, *J* 3.1, 6.1 Hz, *H*3 or *H*4), 7.37–7.42 (m, *H*3 or *H*4 and *H*7), 7.58 (t, *J* 7.8 Hz, *H*8). ^13^C{^1^H} NMR (21.6 °C, CDCl_3_): *δ*_C_ 23.92 (s, *C*10), 24.66 (s, *C*10), 28.94 (s, *C*9), 112.02 (s, *C*3), 124.66 (s, *C*2), 125.41 (s, *C*8), 131.07 (s, *C*4 or *C*7), 131.21 (s, *C*4 or *C*7), 134.52 (s, *C*6), 146.45 (s, *C*5), 181.68 (s, *C*1).

#### 2.1.4 [AuCl(IF^3^)] [5]

The complex [AuCl(IF^3^)] was prepared by reaction of a solution of HIF^3+^Cl^−^ [5], [9] (0.10 g, 0.274 mmol) in 15 mL of acetone with [AuCl(THT)] [7], [8] (0.132 g, 0.411 mmol) and K_2_CO_3_ (0.189 g, 1.37 mmol). Workup as described above for [AuCl(BIPr)] [5] afforded a white powder. Yield 0.053 g (34.7%).

Anal. Calcd for C_15_H_6_N_2_ClF_6_Au or [AuCl(IF^3^)]: C, 32.14; H, 1.08; N, 5.00. Found: C, 31.28; H, 0.43; N, 4.62%. IR (KBr, cm^−1^): 1616 (vs), 1557 (w), 1523 (vs), 1458 (s), 1397 (w), 1364 (m), 1286 (w), 1251 (w), 1178 (m), 1129 (vs), 1104 (m), 1046 (vs), 999 (s), 978 (w), 847 (s), 768 (vw), 756 (w), 736 (w), 724 (vw), 695 (w), 657 (m), 621 (w), 509 (w), 446 (vw). ^1^H NMR (22.1 °C, CDCl_3_): *δ*_H_ 6.91–6.96 (m, *H*5), 7.29 (s, *H*2). ^13^C{^1^H} NMR (21.5 °C, CDCl_3_): *δ*_C_ 101.88 (ddd, *J* = 3.9, 25.0, 50.1 Hz, *C*6), 112.84 (ddd, *J* = 5.4, 15.8, 31.5 Hz, *C*3), 123.39 (s, *C*2), 156.91 (dd, *J* = 5.3, 30.5 Hz, *C*4), 159.47 (dd, *J* = 5.0, 30.5 Hz, *C*4), 162.02 (dd, *J* = 14.4, 28.5 Hz, *C*5), 164.57 (dd, *J* = 14.4, 28.8 Hz, *C*5), 178.61 (s, *C*1).

#### 2.1.5 [AuCl(I^t^Bu)] [5,10]

The complex [AuCl(I^t^Bu)] was prepared by reaction of a solution of HI^t^Bu^+^Cl^−^ [10], [11] (0.400 g, 1.845 mmol) in 60 mL of acetone with [AuCl(THT)] [7], [8] (0.887 g, 2.767 mmol) and K_2_CO_3_ (1.275 g, 9.225 mmol). Workup as described above for [AuCl(BIPr)] [5] afforded a white powder. Yield 0.381 g (50.0%).

Anal. Calcd for C_11_H_20_N_2_ClAu or [AuCl(I^t^Bu)]: C, 32.01; H, 4.88; N, 6.79. Found: C, 32.32; H, 4.60; N, 6.64%. IR (KBr, cm^−1^): 1647 (w), 1559 (w), 1542 (m), 1518 (w), 1507 (w), 1473 (w), 1458 (w), 1438 (w), 1406 (m), 1378 (s), 1305 (w), 1236 (w), 1209 (vs), 1183 (s), 1156 (w), 1053 (vw), 1039 (vw), 1022 (vw), 981 (vw), 962 (vw), 931 (vw), 823 (vw), 720 (m), 693 (s), 626 (w), 418 (vw). ^1^H NMR (21.5 °C, CDCl_3_): *δ*_H_ 1.88 (18H, s, *H*4), 7.11 (2H, s, *H*2). ^13^C{^1^H} NMR (21.4 °C, CDCl_3_): *δ*_C_ 31.76 (s, *C*4), 58.97 (s, *C*3), 116.43 (s, *C*2), 168.03 (s, *C*1).

### 2.2 Preparation of [Au(*RS*-pyrrld)(NHC)] complexes (NHC = IMes (**6**), BIPr (**7**), IF^3^ (**8**), I^t^Bu (**9**))

The [Au(*RS*-pyrrld)(NHC)] complexes (NHC = IMes (**6**), BIPr (**7**), IF^3^ (**8**), I^t^Bu (**9**)) were prepared by reaction of [AuCl(NHC)] with _∞_{[Ag (*RS*-pyrrld)]_2_} [12]. The ^1^H NMR and IR spectra of [Au (*RS*-pyrrld)(IPr)] (**1**) [1] are shown in Fig. 1, Fig. 2.

#### 2.2.1 [Au(*RS*-pyrrld)(IMes)] (6)

Compound (**6**) was prepared by reaction of [AuCl(IMes)] (0.403 g, 0.750 mmol) with _∞_{[Ag(*RS*-pyrrld)]_2_} (0.533 g, 1.13 mmol). Workup as described above for [Au(*RS*-pyrrld)(IPr)] (**1**) afforded a pale yellow powder. Yield 0.119 g (50.4%).

Anal. Calcd for C_26_H_30_N_3_O_3_Au or [Au (*RS*-pyrrld)(IMes)]: C, 49.61; H, 4.80; N, 6.68. Found: C, 48.62; H, 6.31; N, 6.53%. TG/DTA under atmospheric conditions: a weight loss of 71.95% due to decomposition at below 500.0 °C was observed with exothermic peaks at 166.5 and 501.0 °C. IR (KBr, cm^−1^): 1686 (vs), 1652 (s), 1488 (s), 1437 (w), 1415 (m), 1378 (m), 1274 (m), 1239 (m), 1035 (vw), 1014 (vw), 930 (vw), 749 (w), 704 (w), 577 (w), 422 (w). ^1^H NMR (20.9 °C, CDCl_3_): *δ*_H_ 2.13 (s, *H*5), 2.20–2.29 (m, C*H*_*2*_ pyrrld), 2.35 (s, *H*8), 3.97–4.01 (m, C*H* pyrrld), 5.79 (s, N*H* pyrrld), 7.02 (s, *H*6), 7.13 (s, *H*2). ^13^C{^1^H} NMR (22.4 °C, CDCl_3_): *δ*_C_ 17.82 (s, *C*5), 21.19 (s, *C*8), 25.21 (s, *C*H_2_CH pyrrld), 30.21 (s, *C*H_2_CO pyrrld), 57.84 (s, *C*H pyrrld), 122.50 (s, *C*2), 129.54 (s, *C*6), 134.57 (s, *C*4 or *C*7), 134.70 (s, *C*4 or *C*7), 139.82 (s, *C*3), 165.38 (s, *C*1), 175.99 (s, *C*OO pyrrld), 177.68 (s, *C*O).

#### 2.2.2 [Au(*RS*-pyrrld)(BIPr)] (7)

Compound (**7**) was prepared by reaction of [AuCl(BIPr)] (0.227 g, 0.338 mmol) with _∞_{[Ag(*RS*-pyrrld)]_2_} (0.319 g, 0.676 mmol). Workup as described above for [Au (*RS*-pyrrld)(IPr)] (**1**) afforded a pale yellow powder. Yield 0.161 g (60.5%).

Anal. Calcd for C_36.2_H_44.2_N_3_O_3_Cl_0.6_Au or [Au(*RS*-pyrrld)(BIPr)]·0.2CHCl_3_: C, 55.21; H, 5.66; N, 5.34%. Found: C, 55.30; H, 5.70; N, 5.16%. TG/DTA under atmospheric conditions: a weight loss of 3.23% due to desorption of 0.2 CHCl_3_ at below 216.3 °C was observed; calcd 3.03% for 0.2 solvated CHCl_3_ molecules. Further, a weight loss of 76.11% due to decomposition was observed at below 500.0 °C with exothermic peaks at 304.3, 310.5, and 325.2 °C. IR (KBr, cm^−1^): 1705 (vs), 1636 (s), 1469 (m), 1398 (m), 1361 (s), 1295 (m), 1266 (m), 1241 (m), 1181 (vw), 1159 (vw), 1147 (vw), 1092 (vw), 1059 (vw), 1007 (vw), 936 (vw), 801 (m), 752 (m), 594 (w), 474 (vw), 432 (vw), 419 (vw). ^1^H NMR (21.0 °C, CDCl_3_): *δ*_H_ 1.10 (d, *J* 6.9 Hz, *H*10), 1.35 (d, *J* 6.8 Hz, *H*10), 2.02–2.25 (m, C*H*_2_ pyrrld), 2.39 (sept, *J* 6.9 Hz, *H*9), 3.95–3.99 (m, C*H* pyrrld), 5.73 (s, N*H* pyrrld), 7.11 (dd, *J* 3.1, 6.0 Hz, *H*3 or *H*4), 7.39–7.43 (m, *H*3 or *H*4 and *H*7), 7.61 (t, *J* 7.8 Hz, *H*8). ^13^C{^1^H} NMR (22.1 °C, CDCl_3_): *δ*_C_ 23.97 (s, *C*10), 24.58 (s, *C*10), 25.30 (s, *C*H_2_CH pyrrld), 29.00 (s, *C*9), 30.29 (s, *C*H_2_CO pyrrld), 57.78 (s, *C*H pyrrld), 112.02 (s, *C*3), 124.70 (s, *C*2), 125.47 (s, *C*8), 131.10 (s, *C*4 or *C*7), 131.18 (s, *C*4 or *C*7), 134.58 (s, *C*6), 146.48 (s, *C*5), 174.47 (s, *C*1), 175.53 (s, *C*OO pyrrld), 177.54 (s, *C*O).

#### 2.2.3 [Au(*RS*-pyrrld)(IF^3^)] (8)

Compound (**8**) was prepared by reaction of [AuCl(IF^3^)] (0.210 g, 0.374 mmol) with _∞_{[Ag(*RS*-pyrrld)]_2_} (0.356 g, 0.755 mmol). Workup as described above for [Au(*RS*-pyrrld)(IPr)] (**1**) afforded a white powder. Yield 0.117 g (46.2%).

Anal. Calcd for C_20.2_H_12.2_N_3_O_3_Cl_0.6_Au or [Au(*RS*-pyrrld)(IF^3^)]·0.1CHCl_3_: C, 36.29; H, 1,83; N, 6.32. Found: C, 36.06; H, 1.53; N, 6.07%. TG/DTA under atmospheric conditions: a weight loss of 1.04% due to desorption of 0.1 CHCl_3_ at below 188.1 °C was observed; calcd 1.79% for 0.1 solvated CHCl_3_ molecules. Further, a weight loss of 66.79% due to decomposition was observed at below 500.0 °C with exothermic peaks at 201.4, 228.1, and 502.3 °C. IR (KBr, cm^−1^): 1694 (vs), 1648 (vs), 1619 (vs), 1525 (vs), 1459 (s), 1394 (m), 1365 (s), 1261 (m), 1179 (m), 1130 (vs), 1104 (m), 1049 (vs), 1000 (s), 845 (m), 740 (w), 697 (w), 668 (w), 657 (w), 621 (w), 510 (w), 448 (vw). ^1^H NMR (22.1 °C, CDCl_3_): *δ*_H_ 2.10–2.40 (m, C*H*_2_ pyrrld), 4.05–4.09 (m, C*H* pyrrld), 5.77 (s, N*H* pyrrld), 6.94–7.00 (m, *H*5), 7.34 (s, *H*2). ^13^C{^1^H} NMR (21.2 °C, CDCl_3_): *δ*_C_ 25.36 (s, *C*H_2_CH pyrrld), 30.10 (s, *C*H_2_CO pyrrld), 57.73 (s, *C*H pyrrld), 101.98 (ddd, *J* 4.0, 25.0, 50.1 Hz, *C*6), 112.56–112.92 (m, *C*3) 123.58 (s, C2), 156.80 (dd, *J* 5.0, 14.9 Hz, *C*4), 159.39 (dd, *J* 5.0, 17.9 Hz, *C*4), 162.03 (dd, *J* 14.4, 28.9 Hz, *C*5), 164.61 (dd, *J* 14.31, 28.6 Hz, *C*5), 171.38 (s, C1), 176.31 (s, *C*OO pyrrld), 177.70 (s, *C*O).

#### 2.2.4 [Au(*RS*-pyrrld)(I^t^Bu)] (9)

Compound (**9**) was prepared by reaction of [AuCl(I^t^Bu)] (0.299 g, 0.724 mmol) with _∞_{[Ag(*RS*-pyrrld)]_2_} (0.683 g, 1.448 mmol). Workup as described above for [Au(*RS*-pyrrld)(IPr)] (**1**) afforded a white powder. Yield 0.262 g (71.5%).

Anal. Calcd for C_16_H_26_N_3_O_3_Au or [Au (*RS*-pyrrld)(I^t^Bu)]: C, 38.03; H, 5.19; N, 8.31. Found: C, 38.03; H, 5.10; N, 8.00%. TG/DTA under atmospheric conditions: a weight loss of 61.46% due to decomposition at below 500.0 °C was observed with an exothermic peak at 250.6 °C. IR (KBr, cm^−1^): 1697 (vs), 1653 (s), 1604 (s), 1473 (m), 1457 (m), 1406 (s), 1378 (vs), 1305 (m), 1262 (m), 1234 (s), 1213 (vs), 1147 (w), 1038 (vw), 979 (vw), 930 (vw), 825 (vw), 731 (w), 697 (m), 631 (w), 568 (vw), 418 (vw). ^1^H NMR (21.8 °C, CDCl_3_): *δ*_H_ 1.90 (s, *H*4), 2.33–2.50 (m, C*H*_2_ pyrrld), 4.24–4.28 (m, C*H* pyrrld), 5.90 (s, N*H* pyrrld), 7.11 (s, *H*2). ^13^C{^1^H} NMR (22.0 °C, CDCl_3_): *δ*_C_ 25.58 (s, *C*H_2_CH pyrrld), 30.29 (s, *C*H_2_CO pyrrld), 31.70 (s, *C*4), 57.83 (s, *C*H pyrrld), 59.12 (s, *C*3), 116.67 (s, *C*2), 159.66 (s, C1), 176.68 (s, *C*OO pyrrld), 177.77 (s, *C*O pyrrld).

### 2.3 X-ray crystallography of [Au(IPr)(H_2_O)]_3_[α-PW_12_O_40_]·7Et_2_O (**2**)

Crystallization of (**2**), whose ^1^H NMR spectrum is shown in Fig. 3 [1], was carried out by liquid-liquid diffusion of an internal aqueous solution of the metal complex with an external solvent (ether) in a refrigerator. Single crystals of the metal complex were mounted on a loop and used for measurements of cell constants and for the collection of intensity data on a Rigaku VariMax with Saturn CCD diffractometer. The structure was solved by a direct method, followed by difference Fourier calculation; it was refined by a full-matrix least-squares method on *F*^2^ using the Yadokari program package [13]. All non-hydrogen atoms were refined anisotropically. The hydrogen atoms were placed geometrically or identified on a difference Fourier-map and were treated using a riding model. The crystal data of (**2**) are summarized in Table 1, and selected bond distances (Å) and angles (deg) are shown in Table 2. The details of the crystal data have been deposited with the Cambridge Crystallographic Data Centre as a supplementary publication (CCDC no. 1864226).
