# Supplementary material for: Dataset of polyoxometalate-assisted N-heterocyclic carbene gold(I) complexes
Source: Data Brief. 2019 May 24;25:104002. doi: 10.1016/j.dib.2019.104002 (PMC6557754; doi:10.1016/j.dib.2019.104002)
Supplement: Multimedia component 1 [file mmc1.doc]

Conflict of Interest and Authorship Conformation Form

Please check the following as appropriate:

- All authors have participated in (a) conception and design, or analysis and interpretation of the data; (b) drafting the article or revising it critically for important intellectual content; and (c) approval of the final version.
- This manuscript has not been submitted to, nor is under review at, another journal or other publishing venue.
- The authors have no affiliation with any organization with a direct or indirect financial interest in the subject matter discussed in the manuscript
- The following authors have affiliations with organizations with direct or indirect financial interest in the subject matter discussed in the manuscript:

Author’s name Affiliation

Kenji Nomiya Department of Chemistry, Faculty of Science, Kanagawa University

Yuichi Murara Department of Chemistry, Faculty of Science, Kanagawa University

Yuta Iwasaki Department of Chemistry, Faculty of Science, Kanagawa University

Hidekazu Arai Department of Chemistry, Faculty of Science, Kanagawa University

Takuya Yoshida Department of Chemistry, Faculty of Science, Kanagawa University

Noriko Chikaraishi Kasuga Department of Chemistry, Faculty of Science, Kanagawa University

Toshiaki Matsubara Department of Chemistry, Faculty of Science, Kanagawa University
